# Pharmacological Mechanisms Underlying the Anti-asthmatic Effects of Modified Guomin Decoction Determined by Network Pharmacology and Molecular Docking

**DOI:** 10.3389/fmolb.2021.644561

**Published:** 2021-04-22

**Authors:** Guishu Wang, Bo Zhou, Zheyi Wang, Yufeng Meng, Yaqian Liu, Xiaoqin Yao, Cuiling Feng

**Affiliations:** ^1^Dongzhimen Hospital Affiliated to Beijing University of Chinese Medicine, Beijing, China; ^2^Department of TCM, Peking University People’s Hospital, Beijing, China; ^3^Xiyuan Hospital Affiliated to China Academy of Chinese Medical Sciences, Beijing, China; ^4^Department of TCM, Peking University International Hospital, Beijing, China; ^5^Department of Integration of Chinese and Western Medicine, School of Basic Medical Sciences, Peking University, Beijing, China

**Keywords:** asthma, airway inflammation, airway remodeling, Chinese medicine, network pharmacology

## Abstract

**Background:**

Asthma is a chronic inflammatory disease characterized by Th2-predominant inflammation and airway remodeling. Modified Guo Min decoction (MGMD) has been an extensive practical strategy for allergic disorders in China. Although its potential anti-asthmatic activity has been reported, the exact mechanism of action of MGMD in asthma remains unexplored.

**Methods:**

Network pharmacology approach was employed to predict the active components, potential targets, and molecular mechanism of MGMD for asthma treatment, including drug-likeness evaluation, oral bioavailability prediction, protein–protein interaction (PPI) network construction and analysis, Gene Ontology (GO) terms, and Reactome pathway annotation. Molecular docking was carried out to investigate interactions between active compounds and potential targets.

**Results:**

A total of 92 active compounds and 72 anti-asthma targets of MGMD were selected for analysis. The GO enrichment analysis results indicated that the anti-asthmatic targets of MGMD mainly participate in inflammatory and in airway remolding processes. The Reactome pathway analysis showed that MGMD prevents asthma mainly through regulation of the IL-4 and IL-13 signaling and the specialized pro-resolving mediators (SPMs) biosynthesis. Molecular docking results suggest that each bioactive compounds (quercetin, wogonin, luteolin, naringenin, and kaempferol) is capable to bind with STAT3, PTGS2, JUN, VEGFA, EGFR, and ALOX5.

**Conclusion:**

This study revealed the active ingredients and potential molecular mechanism by which MGMD treatment is effective against airway inflammation and remodeling in asthma through regulating IL-4 and IL-13 signaling and SPMs biosynthesis.

## Introduction

Asthma is a globally prevalent chronic inflammatory pulmonary disease, with an estimated 358 million affected individuals (Soriano et al., 2017). Persistent airway inflammation is the hallmark of asthma, which is infiltrated eosinophils, T lymphocytes, mast cells, and release of pro-inflammatory cytokines and lipid mediators. Lipid mediators, such as specialized pro-resolving mediators (SPMs), play a decisive role in the resolution of inflammation in asthma (Schett and Neurath, 2018; Kytikova et al., 2019). Actions of SPMs generated from polyunsaturated fatty acids (PUFAs) include inhibition of eosinophil and neutrophil migration, and decrease of T helper 2 (Th2) cytokine production (e.g., IL-4, IL-5, and IL-13) (Barnig et al., 2018). SPMs are originated via lipoxygenase (LOX)–catalyzed reaction. SPMs include omega-6 PUFA arachidonic acid derived lipoxins, as well as omega-3 PUFA eicosapentaenoic acid (EPA) and docosahexaenoic acid (DHA) derived resolvins, protectins and maresins. Arachidonic acid also forms a range of pro-inflammatory mediators in asthma, such as prostaglandins via cyclooxygenase (COX)-2, and the leukotrienes via 5-LOX actions (Krishnamoorthy et al., 2018).

Airway remodeling is another key pathological features of asthma. It is characterized by thickening of basement membrane, goblet cell metaplasia, smooth muscle cell hyperplasia and hypertrophy, deposition of extracellular matrix proteins, vascular activation and angiogenesis (Jeffery, 2001; James and Wenzel, 2007). Synthesis of extracellular matrix components and collagen is promoted by epidermal growth factor (EGF) released by airway epithelial cells and TGF-β produced by myofibroblasts (Flood-Page et al., 2003; Le Cras et al., 2011). Under the influence of airway epithelial cells-derived endothelial growth factor (VEGF)-A, angiogenesis allow for extravasation of more inflammatory cells (Lambrecht and Hammad, 2012).

**FIGURE 1:**
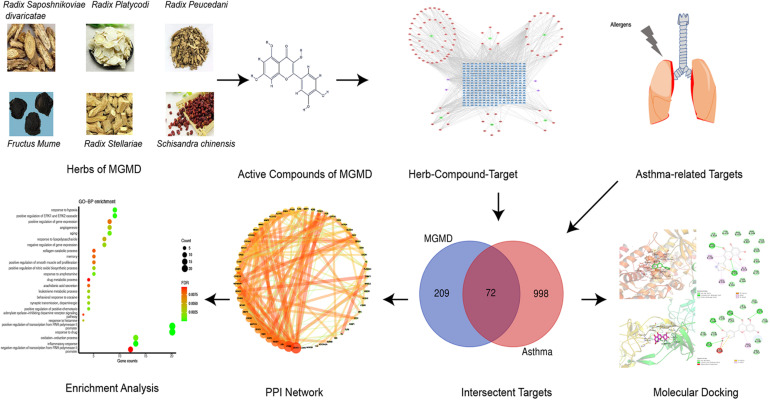
Flowchart summarizing the work scheme of the study.

Traditional Chinese medicine (TCM) has been used to treat allergic diseases in Asia for hundreds of years (Liu L. et al., 2018). “Guo Min” means allergy in Chinese and Guo Min decoction (GMD) is a compound recipe composed by herbs, that raised by a well-known Chinese physician, Zhu Shenyu (Zhu and Liang, 1985). GMD has been widely used for allergic diseases treatment in China (Tao et al., 2018). The Modified Guo Min decoction (MGMD) comprises Fang Feng (*Radix Saposhnikoviae divaricate*, FF), Wu Mei (*Fructus Mume*, WM), Wu Wei Zi (*Schisandra chinensis*, WWZ), Yin Chai Hu (*Radix Stellariae*, YCH), Qian Hu (*radix Peucedani*, QH), and Jie Geng (*Radix Platycodi*, JG). Previous studies showed that prescriptions based on GMD increased the proportion of Th1 cells in the spleen tissue of asthmatic mice, reduced the Th2 subset of T cells, and balanced the number of Treg and Th17 cells (Qin et al., 2017). However, the therapeutic mechanisms of MGMD in asthma remain unclear. In this study, network pharmacology approach was used to explore the multi-components, multi-targets, and multi-pathways closely related to the anti-asthma effect of MGMD. The molecular docking method was also used to verify the binding of the active substance to the target of action to reveal and predict the efficacy of MGMD in asthma. The work scheme is shown in Figure 1.

## Materials and Methods

### Active Compounds Collection

The compounds of MGMD were extracted from the HERB database^1^ (Fang et al., 2020) and the encyclopedia of traditional Chinese medicine (ETCM)^2^ (Xu et al., 2019). HERB contains a comprehensive list of 7263 TCM herbs and 49258 ingredients by integrating multiple TCM databases included SymMap (Wu et al., 2019), TCMID 2.0 (Huang et al., 2018), TCMSP 2.3 (Ru et al., 2014), and TCM-ID (Chen et al., 2006). Oral bioavailability (OB) ≥ 30% and drug-likeness (DL) ≥ 0.18 were set as the thresholds to screen the active compounds from the TCMSP^3^. The properties of the other ingredients collected from the HERB were retrieved from the Swiss absorption, distribution, metabolism, and excretion (ADME) database^4^. The SMILES format files of the compounds were obtained from the PubChem^5^ database (Kim et al., 2019). The screening criterion for gastrointestinal GI absorption was set as high, and DL was satisfied with both “yes” at the same time (Daina et al., 2017). Active ingredients from ETCM were retrieved as DL Grading “Good” (Xu et al., 2019).

### Target Fishing

#### Identification of Predicted Targets of MGMD

The active ingredients in drugs exert their biological effects via molecular targets. The targets related to the active compounds presented in MGMD were searched from ETCM (Xu et al., 2019) and Similarity ensemble approach (SEA)^6^ (Keiser et al., 2007). ETCM predicts targets by MedChem Studio and only outputs these targets with confidence score at least 0.8. The threshold of “human” and Tanimoto coefficients (Tc) > 0.57 were set to filter results predicted by SEA. The key targets corresponding to the compounds in such six herbs were obtained.

#### Building the Disease Target Database

Asthma-related targets were identified by integrating data from these databases: Comparative Toxicogenomics Database (CTD)^7^ (Grondin et al., 2018), Online Mendelian Inheritance in Man (OMIM)^8^ (Amberger and Hamosh, 2017), and the Human Gene Database (GeneCards)^9^ (Safran et al., 2010). For filtering the targets, “asthma” was defined as the key word and results were restricted to human genes/proteins. Targets with a relevance score above the median were selected in GeneCards database. The overlapping targets between MGMD and asthma were likely to be potential action targets of MGMD in the treatment of asthma.

### PPI Network Construction and Analysis

To obtain the data of protein–protein interaction (PPI), the candidate targets were inputted to STRING^10^ with a minimum required interaction score > 0.7 and limited to “Homo sapiens” specie (von Mering et al., 2005). The visual network graphs were created by Cytoscape (version 3.7.1)^11^, an open-source software platform for visualizing complex networks (Shannon et al., 2003). The target interaction network parameters were calculated by NetworkAnalyzer.

Molecular Complex Detection (MCODE) of Cytoscape was used to searching the highly connected sub-networks in the PPI network. In the illustrations depicting MCODE results, a vertex-weighting-based scheme was used to identify local high-density areas in the graph. Density was generally defined based on the connection level of the graph (Bader and Hogue, 2003).

### Protein Functional Enrichment Analysis

To reveal the potential biological functions of MGMD in the treatment of asthma, functional enrichment analyses were performed by the Database for Annotation, Visualization and Integrated Discovery (DAVID, v6.8)^12^ and Reactome^13^ (Fabregat et al., 2018). The results for Gene ontology (GO) biological processes (BPs) and Reactome pathway enrichment were saved and sorted by the adjusted *P* value corrected by the false discovery rate (FDR) algorithm for each term.

### Network Construction

To demonstrate the multi-compound therapeutic features of MGMD, network constructions were performed as follows: (1) herb-compound-target Network (H-C-T network) was constructed to explore the active compounds and their potential targets. The core compounds were obtained through the H-C-T network. (2) PPI networks were built to analyze the target interactions. Hub targets involved in MGMD treatment of asthma were selected from the PPI network. (3) BP sub-networks were established for classification analysis of BPs in MGMD treatment for asthma. (4) Target pathway network (T-P network) was constructed to show the functional pathways of MGMD for the therapy of asthma.

### Molecular Docking

Molecular docking was conducted to validate if MGMD’s compounds could bind to these targets. The 2D structures of the top five core compounds were downloaded from the TCMSP database (Ru et al., 2014). The structures were added charge and displayed rotatable keys by AutoDock Tools (version 1.5.6). The protein crystal structures corresponding to the core target genes were downloaded from the Protein Data Bank database (PDB)^14^ (Burley et al., 2017). Water and hetero molecules of the proteins were removed by Pymol. Hydrogen atoms and charge operations to the proteins was added by AutoDock Tools. The 3D Grid box for molecular docking simulation was also obtained by AutoDock tools was displayed by AutoDock Vina (version 1.1.2) (Trott and Olson, 2010). The results were analyzed and interpreted by PyMOL and Discovery Studio 2020.

## Results

### Construction of Herb-Compound-Target Network

In this study, 96 active compounds were screened from the six herbs in MGMD. Among them, 51, 19, 7, 6, 8, and 5 compounds were from FF, QH, JG, WM, WWZ, and YCH, respectively. MGMD contains a complex mixture of ingredients, some of them overlapped across 2 herbs, including decursinol, deoxygomisin A, nodakenetin, and naringenin. A total of 92 active compounds were identified after eliminating redundant entries.

Five hundred and twenty-three targets were associated with the 92 components identified in MGMD, of which 149 were associated with FF, 151 with QH, 83 with JG, 77 with WM, 23 with WWZ, and 40 with YCH. After eliminating overlapping targets, there were 281 targets remaining.

**FIGURE 2:**
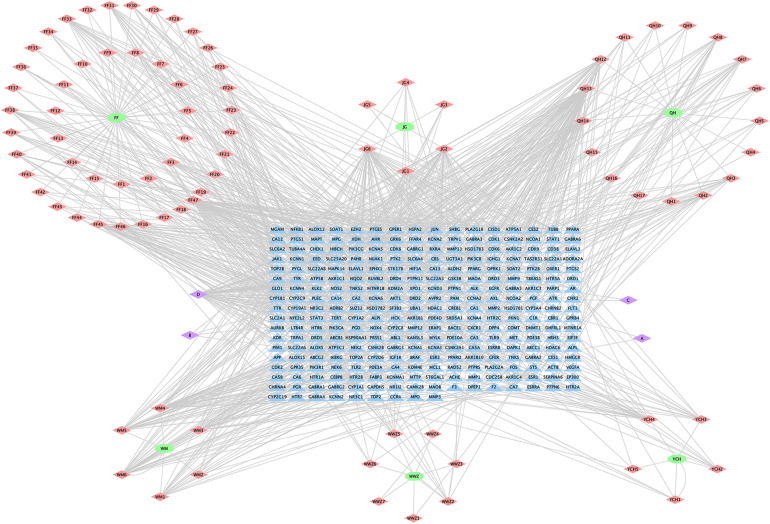
Herb-Compound-Target network (H-C-T network) of MGMD. Green ellipses represent the herbs present in MGMD; pink diamonds represent active compounds in each herb; purple diamonds represent active compounds shared by two herbs, and blue triangles correspond to related targets (The IDs of the components are described in Supplementary Table 1).

The H-C-T network of MGMD was visualized in Cytoscape (Figure 2). The network contained 379 nodes and 1021 edges. Quercetin showed the highest degree of connectivity in the network with 76 targets, followed by wogonin with 57, luteolin with 55, naringenin with 51, and kaempferol with 40. The properties of the H-C-T network were suitable for displaying complex ingredients, multiple targets, and close interactions between ingredients and targets. Detailed information about the active compounds and targets identified in MGMD is shown in Supplementary Table 1.

### Potential Asthma Targets

The targets for asthma were integrated from multi-source databases and a final list of 1,070 disease-related targets obtained after eliminating duplicates (Supplementary Table 2). 72 overlapping targets were identified as the key targets for studying the anti-asthmatic activity of the MGMD compounds (Supplementary Table 3).

### Analysis of the Network of Overlapping Targets

#### Protein–Protein Interaction (PPI) Network

**FIGURE 3:**
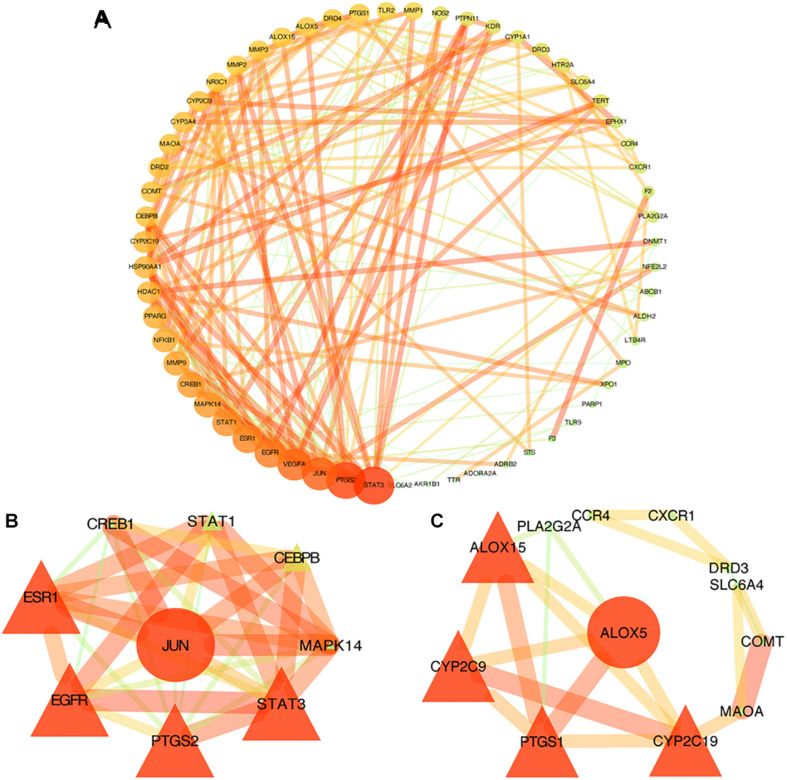
PPI networks of MGMD for asthma treatment. Each node represents a protein target and each line represents the interaction between two nodes. Nodes in red are important and nodes in green are less important in the network. Panel **(A)** is the PPI network diagram arranged according to the df. Two clusters detected in the MGMD-asthma PPI network. Panels **(B,C)** show clusters 1 and 2, respectively. The ellipses are seed nodes of each cluster.

The STRING database was used to acquire PPI relationships of 72 potential protein targets of MGMD as related to the treatment of asthma. The visualized PPI network was constructed by Cystoscape 3.7.1, constructing by 72 nodes represented proteins and 205 edges represented the interactions between the proteins (Figure 3A and Supplementary Table 4). The average node degree value of the PPI network was 6.508. 28 protein targets had significantly higher node degree than the average in the PPI network, including STAT3, PTGS2, JUN, vascular endothelial growth factor A (VEGFA), EGFR, ESR1, STAT1, CREB1, MAPK14, MMP9, NFKB1, PPARG, CYP2C19, HDAC1, HSP90AA1, CYP3A4, COMT, MAOA, CYP2C9, DRD2, CEBPB, ALOX15, PTGS1, arachidonate 5-lipoxygenase (ALOX5), DRD4, NR3C1, MMP3, and MMP2. These 28 targets showed the preliminary relationships between active compound and potential protein targets of MGMD treatment in asthma.

#### Clustering Analysis of the PPI Network

Similar function clusters of the PPI network were selected by MCODE analysis based on topology to find densely interconnected regions using Cytoscape software. 2 clusters of functional modules were detected (K-core = 4) and the attribute values of the cluster were shown in Figures 3B,C.

Clusters in a PPI network are often protein complexes and parts of pathways, while clusters in a protein similarity network represent protein families. Cluster 1 comprised nine nodes and 33 edges with a score of 8.250 (Figure 3B). The seed node of this cluster was JUN (Jun proto-oncogene, also known as AP-1, cJUN, c-Jun), which involved in cell proliferation, differentiation, migration, transformation, and programmed cell death (Liu and Lin, 2007). Cluster 2 comprised 12 nodes and 21 sides with a score of 3.818 (Figure 3C). The seed node of this cluster was ALOX5 (arachidonate 5-lipoxygenase, also known as 5-LO, 5-LOX), an essential enzyme in the metabolism of arachidonic acid, which initiates the biosynthesis of leukotrienes (Bruno et al., 2018). Leukotrienes are powerful immune-regulating lipid mediators with established pathogenic roles in asthma. The connected proteins of ALOX5 in cluster 2 participating in the biosynthetic pathways of SPMs (Barnig et al., 2018; Kytikova et al., 2019), such as ALOX15 (15-LOX), cytochrome P450 proteins CYP2C9 and CYP2C19, are among the 28 protein targets which had higher node degree than the average in our PPI network. Thus, the constructed PPI network and the further functional clustering both indicate that MGMD intervene asthma by regulating SPMs biosynthesis.

### GO Enrichment Analysis

**FIGURE 4:**
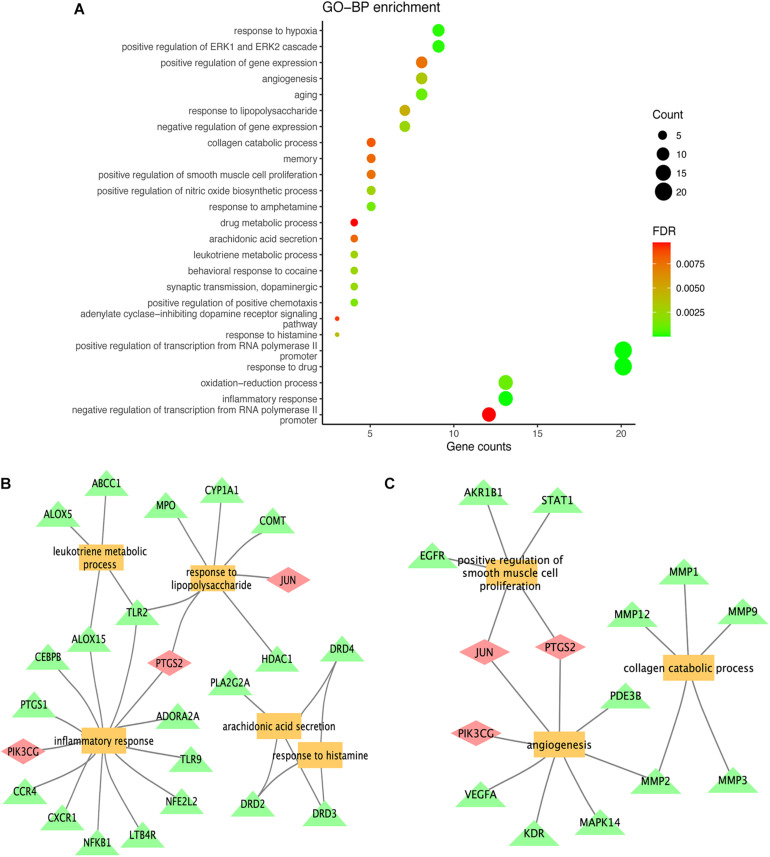
Results of GO term enrichment analysis of targets. The GO bar plot represents the top 25 biological (BP) process **(A)**; inflammation and airway remodeling related to functional clusters represented by panels **(B,C)**, respectively. The yellow squares represent biological processes, the green triangles represent genes, and the pink diamonds represent genes common to both categories.

To further explore the biological functions of the 72 potential targets of MGMD, GO enrichment analysis was constructed on DAVID website and obtained 76 terms (FDR < 0.05) of BPs. We found the top 25 most enriched BP terms represented in a bubble chart (Figure 4A and Supplementary Table 5). BP analysis revealed that the potential targets of MGMD were remarkably enriched in inflammation and airway remodeling.

As shown in Figure 4B, the potential targets of MGMD were involved in inflammatory response (GO ID: 0006954), response to lipopolysaccharide (GO ID: 0032496), leukotriene metabolic process (GO ID: 0006691), arachidonic acid secretion (GO ID: 0050482) and positive regulation of nitric oxide biosynthetic process (GO ID: 0045429). In addition, the BPs of airway remodeling were mainly enriched in positive regulation of smooth muscle cell proliferation (GO ID: 0048661), angiogenesis (GO ID: 0001525) and collagen catabolic process (GO ID: 0030574) (Figure 4C).

### Pathway Enrichment Analysis

**FIGURE 5:**
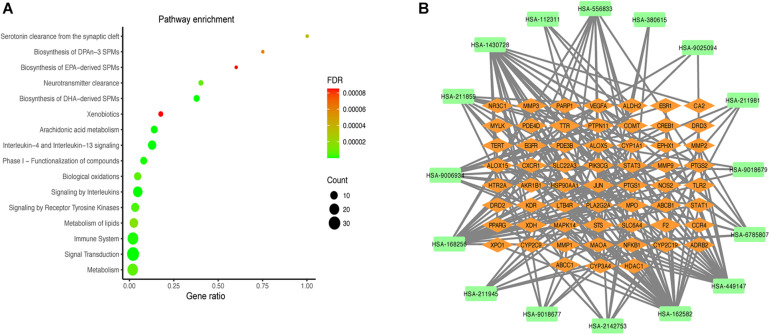
Results of the pathway analysis of the top 16 pathways: Bubble diagram of pathway **(A)** and T-P network diagram **(B)**.

Reactome pathway enrichment analysis obtained 123 items (FDR < 0.05) and the top 16 pathways at a significance threshold of FDR < 10^–5^ were displayed (Figure 5A and Table 1). The pathways result was intensively enriched in SPMs biosynthesis and inflammatory and immune response, including arachidonic acid metabolism, metabolism of lipids, biosynthesis of EPA-derived SPMs, biosynthesis of DHA-derived SPMs, biosynthesis of DPAn-3 SPMs, interleukin-4 and interleukin-13 signaling, and signaling by interleukins and immune system.

**TABLE 1:** Information on enrichment analysis based on Reactome.

Term ID	Pathway	Counts	FDR	Genes
HSA-6785807	Interleukin-4 and Interleukin-13 signaling	13	2.47E-13	MMP2, STAT3, MMP3, MMP1, NOS2, HSP90AA1, MAOA, STAT1, PTGS2, MMP9, ALOX5, ALOX15, VEGFA
HSA-449147	Signaling by Interleukins	18	1.16E-11	MMP2, NFKB1, MAPK14, STAT3, MMP3, MMP1, NOS2, HSP90AA1, MAOA, PTPN11, STAT1, PTGS2, JUN, MMP9, ALOX5, CREB1, ALOX15, VEGFA
HSA-162582	Signal Transduction	33	4.72E-09	NFKB1, MAPK14, NR3C1, TTR, KDR, STAT3, EGFR, PDE3B, PPARG, CXCR1, MMP3, ADRB2, F2, TERT, CCR4, HSP90AA1, PTPN11, PDE4D, PIK3CG, MYLK, STAT1, DRD2, PARP1, JUN, MMP9, HDAC1, DRD3, LTB4R, XPO1, CREB1, ESR1, HTR2A, VEGFA
HSA-2142753	Arachidonic acid metabolism	8	1.49E-08	CYP2C9, PTGS1, PTGS2, CYP2C19, ALOX5, CYP1A1, ABCC1, ALOX15
HSA-9018677	Biosynthesis of DHA-derived SPMs	6	1.49E-08	CYP2C9, CYP3A4, PTGS2, ALOX5, CYP1A1, ALOX15
HSA-211945	Phase I - Functionalization of compounds	8	6.39E-07	CYP2C9, ALDH2, CYP3A4, MAOA, PTGS1, CYP2C19, CYP1A1, EPHX1
HSA-168256	Immune System	25	7.37E-07	MMP2, MPO, NFKB1, MAPK14, TTR, TLR2, STAT3, CXCR1, MMP3, F2, MMP1, NOS2, HSP90AA1, MAOA, PTPN11, STAT1, PTGS2, JUN, MMP9, ALOX5, XDH, PLA2G2A, CREB1, ALOX15, VEGFA
HSA-9006934	Signaling by Receptor Tyrosine Kinases	12	4.85E-06	MAPK14, KDR, STAT3, EGFR, PDE3B, HSP90AA1, PTPN11, STAT1, MMP9, CREB1, ESR1, VEGFA
HSA-211859	Biological oxidations	9	6.89E-06	CYP2C9, ALDH2, CYP3A4, MAOA, COMT, PTGS1, CYP2C19, CYP1A1, EPHX1
HSA-1430728	Metabolism	24	7.31E-06	STS, TTR, CYP2C9, ALDH2, SLC22A3, CA2, AKR1B1, PPARG, HSP90AA1, CYP3A4, MAOA, PIK3CG, COMT, PTGS1, PTGS2, CYP2C19, ALOX5, XDH, CYP1A1, ABCC1, PLA2G2A, ALOX15, ABCB1, EPHX1
HSA-112311	Neurotransmitter clearance	4	7.71E-06	SLC6A4, ALDH2, MAOA, COMT
HSA-556833	Metabolism of lipids	14	1.54E-05	STS, CYP2C9, AKR1B1, PPARG, CYP3A4, PIK3CG, PTGS1, PTGS2, CYP2C19, ALOX5, CYP1A1, ABCC1, PLA2G2A, ALOX15
HSA-380615	Serotonin clearance from the synaptic cleft	3	3.63E-05	SLC6A4, ALDH2, MAOA
HSA-9025094	Biosynthesis of DPAn-3 SPMs	3	5.97E-05	PTGS2, ALOX5, ALOX15
HSA-211981	Xenobiotics	4	8.52E-05	CYP2C9, CYP3A4, CYP2C19, CYP1A1
HSA-9018679	Biosynthesis of EPA-derived SPMs	3	8.52E-05	PTGS2, ALOX5, ALOX15

The T-P network showed the associations of the top 16 pathways with 75 nodes and 187 edges (Figure 5B). The results revealed that the active compounds of the MGMD had effects on asthma through regulating various pathways, in particular through improving airway inflammatory infiltration resulted from Th2 cytokine production and lipid mediators.

### Molecular Docking Verification of Core Compounds and Core Protein Targets

Furthermore, we verified the binding affinity between core compounds and protein targets of MGMD by molecular docking. As screened in H-C-T network, quercetin, wogonin, luteolin, naringenin, and kaempferol were the core compounds of MGMD. The hub protein targets were identified by top 5 node degree of PPI network and seed node of clusters, included STAT3, PTGS2, JUN, VEGFA, EGFR, and ALOX5.

**FIGURE 6:**
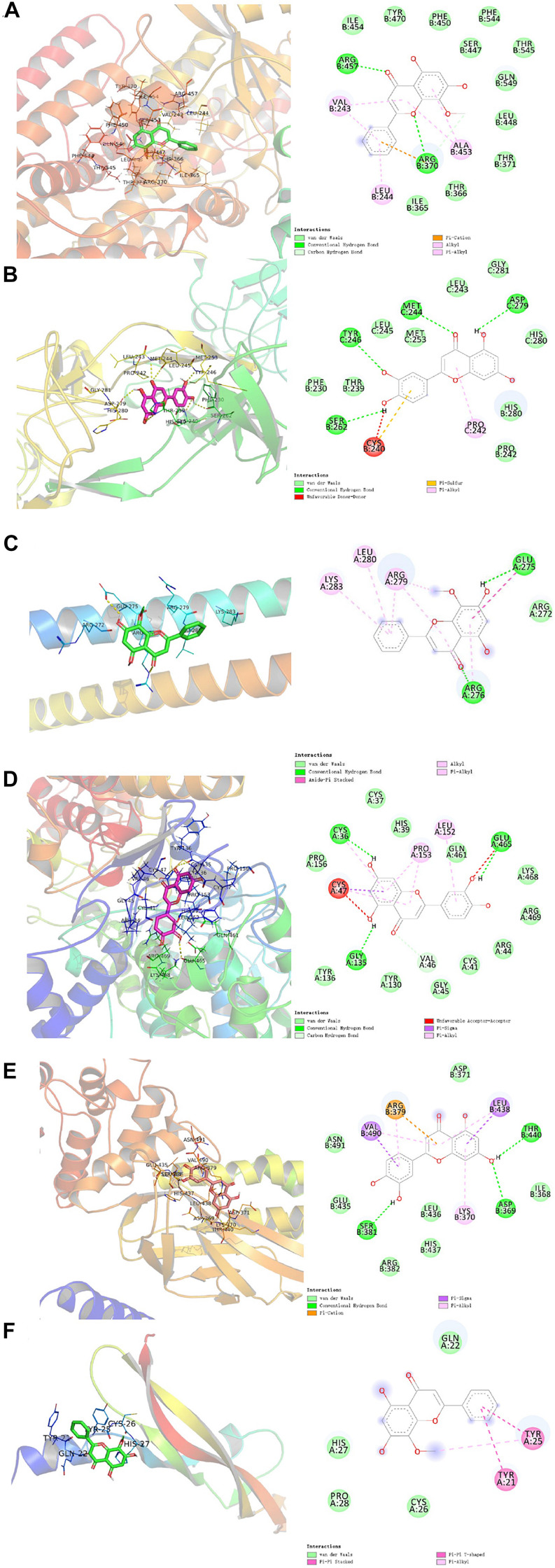
Molecular docking diagram of ALOX5 **(A)**, JUN **(B)**, VEGFA **(C)** complexed with wogonin; EGFR **(D)**, PTGS2 **(E)**, and STAT3 **(F)** complexed with luteolin.

The results obtained by the molecular docking software were shown in Table 2. The Grid box was centered to cover the active binding site and all essential residues. For the STAT3, grid box (126Å × 60Å × 126Å) centered at (0.233, 28.877, 33.555) Å, for the PTGS2, grid box (76Å × 94Å × 90Å) centered at (33.269, −4.946, 7.759) Å, for the JUN, grid box (90Å × 72Å × 70Å) centered at (−16.564, 23.05, 26.225) Å, for the VEGFA, grid box (58Å × 40Å × 40Å) centered at (2.965, −4.946, 7.759) Å, for the EGFR, grid box (126Å × 126Å × 126Å) centered at (80.339, 11.821, 66.594) Å, for the ALOX5, grid box (70Å × 90Å × 96Å) centered at (4.01, 45.829, 3.32) Å. As seen from Table 2, the binding affinity for the five core compounds and protein crystal structures corresponding to the core target genes were all greater than −5 kcal/mol, indicating that the compound had a certain affinity for the protein crystal structure. Wogonin showed the high binding affinity of −8.8 kcal/mol in ALOX5, −6.2 kcal/mol in JUN and −6.2 kcal/mol in VEGFA. Luteolin showed the high binding affinity of −8.3 kcal/mol in EGFR, −9.3 kcal/mol in PTGS2 and −7.9 kcal/mol in STAT3. The small-molecule compounds were tightly bound to the protein residues via various interactions (Figure 6 and Table 2).

**TABLE 2:** The binding energy values of core compounds of MGMD and core targets.

Target	Compounds	binding affinity/(kcal/mol)
STAT3(6tlc)	Quercetin	−7.5
	Wogonin	−7.5
	Luteolin	−7.9
	Naringenin	−7.3
	Kaempferol	−7.7
PTGS2(5ikr)	Quercetin	−9.1
	Wogonin	−8.9
	Luteolin	−9.3
	Naringenin	−9
	Kaempferol	−9
JUN(5T01)	Quercetin	−5.9
	Wogonin	−6.2
	Luteolin	−5.9
	Naringenin	−5.8
	Kaempferol	−6.2
VEGRA(4kzn)	Quercetin	−5.8
	Wogonin	−6.2
	Luteolin	−5.8
	Naringenin	−5.7
	Kaempferol	−6
EGFR(5wb7)	Quercetin	−7.9
	Wogonin	−7.9
	Luteolin	−8.3
	Naringenin	−7.4
	Kaempferol	−7.9
ALOX5(3o8y)	Quercetin	−8.3
	Wogonin	−8.8
	Luteolin	−8.5
	Naringenin	−8.3
	Kaempferol	−8.2

## Discussion

Asthma is a major public health challenge in China that affecting 45.7 million adults aged 20 years or older (Huang et al., 2019). TCM has a long history of clinical practice with available records, which provides a considerable number of classical herbal formulas widely used for disease treatment and represents a unique resource for drug development (Chao et al., 2017). Among the complex mixtures of compounds present in herbal medicines, unique combinations in the traditional complex formulas may exert major efficacy by targeting specific nodes of cellular signaling to intervene a specific chronic disease (Stermitz et al., 2000). Network pharmacology is used to study the intervention mechanism of drugs on diseases by constructing a biological interaction network (Yang et al., 2013). It systematically reveals the therapeutic effects of drugs on diseases from the interaction relationship between drugs, targets, and disease (Liu J. et al., 2018; Zhang et al., 2018). In a previous clinical study, we have found that MGMD had significant efficacy on respiratory symptoms (Zhang, 2016). To provide further insight of underlying mechanisms, we used the systematic pharmacology method and molecular docking to explore the potential molecular mechanism of the bioactive compounds.

We obtained 92 active compounds of MGMD from HERB, ECTM, SEA databases. A total of 72 putative anti-asthmatic targets of MGMD were selected by overlapping the targets between the MGMD-associated targets and the predicted asthma targets. According the H-C-T network topological analysis, we found the core compounds contained quercetin, wogonin, luteolin, naringenin, and kaempferol. Intriguingly, these compounds have all been reported to have therapeutic effects on inflammatory disease in previous studies. It had been demonstrated that all of the five core ingredients can reduce the allergic airway inflammation, inhibit levels of Th2 cytokine, including IL-4, IL-5, and IL-13, in OVA-sensitized and–challenged animals (Park et al., 2009; Shi et al., 2014; Bui et al., 2017; Jang et al., 2017; Molitorisova et al., 2021). Moreover, quercetin can inhibit human neutrophil elastase-induced MUC5AC expression in human airway epithelial cells through PKC/EGFR/ERK signal transduction pathway (Li et al., 2012). Both wogonin and luteolin had effect on reduction of airway mucus production (Lucas et al., 2015; Wang et al., 2021), meanwhile luteolin can also decrease collagen deposition *in vovo* (Wang et al., 2021). In addition, it had reported that kaempferol alleviated airway inflammation through modulating Tyk2-STAT1/3 signaling responsive (Gong et al., 2013), and suppressed COX2 expression level in lung tissues in asthmatic mice (Kang et al., 2018). These previous studies suggest that MGMD is likely to decrease Th2 cytokine production in asthma mainly through compound of quercetin, wogonin, luteolin, naringenin, and kaempferol.

The PPI network was constructed by 72 potential targets of MGMD which were involved in asthma, and the network was further analyzed by MCODE clustering to explore the asthma related pathways. Our results suggested that the core potential targets of MGMD were intensively enriched in the pathways related to SPMs biosynthesis, including ALOX5 (5-LOX), ALOX15 (15-LOX), PTGS1 (COX1), PTGS2 (COX2), cytochrome P450 proteins CYP2C9 and CYP2C19, which paly pivotal role in the resolution of inflammation in asthma (Schett and Neurath, 2018; Kytikova et al., 2019). 5-LOX is a crucial enzyme which helps in the conversion of arachidonic acid to leukotrienes. With the increasing number of indications for anti-leukotriene drugs, the development of 5-LOX inhibitor agents for asthma becomes increasingly important (Bruno et al., 2018; Sinha et al., 2019). Our results argue a potential that MGMD is a 5-LOX inhibitor for the treatment of asthma, in turn suggest a promising mechanism by which MGMD intervene asthma.

Furthermore, STAT3, JUN, EGFR, and VEGFA were also the core potential targets in our PPI network results. STAT3 and JUN are closely related to the pathological process of asthma (Simeone-Penney et al., 2007; García-Menaya et al., 2019). STAT3 can induce gene expression of cytokines, chemokines, and adhesion molecules with an important role in directing the inflammatory response and act as a novel epithelial regulator of the allergic response by altering Th2 cell recruitment and effector function (Schumann et al., 1996; Simeone-Penney et al., 2007). JUN activation appears to be implicated in airway inflammation by contributing to regulate the expression of Th2 cytokines, chemokines, growth factors, and adhesion molecules, which is associated with several inflammation related disorders such as asthma and allergy (Haeggström and Funk, 2011). There are extensive studies focusing on the relation among EGFR, VEGFA and asthma pathophysiology, which describe airway remodeling, airway hypermucus secretion, as well as immunological responses of airway inflammation (Hoshino et al., 2001; Lee et al., 2004; Burgel and Nadel, 2008; Inoue et al., 2020). MGMD compounds may exert anti-inflammatory and anti-remodeling activity through regulating STAT3, JUN, EGFR, and VEGFA.

The GO enrichment results strengthen the evidence that candidate protein targets of MGMD are involved in airway inflammation and remodeling. The enriched BPs of anti-inflammatory in our results included inflammatory response, leukotriene metabolic process and arachidonic acid secretion, suggesting that MGMD could influenced the lipid mediators, both pro-inflammatory mediators and SPMs, to ameliorate the airway inflammation in asthma patients. Moreover, the typical airway remodeling processes, including positive regulation of smooth muscle cell proliferation, angiogenesis and collagen catabolic process, were also enriched by MGMD related targets. Combined with the PPI network results, we propose that MGMD regulates SPMs biosynthesis by ALOX5 to reduce airway inflammation and relieve airway remodeling process by suppressing the EGFR and VEGFA signaling.

**FIGURE 7:**
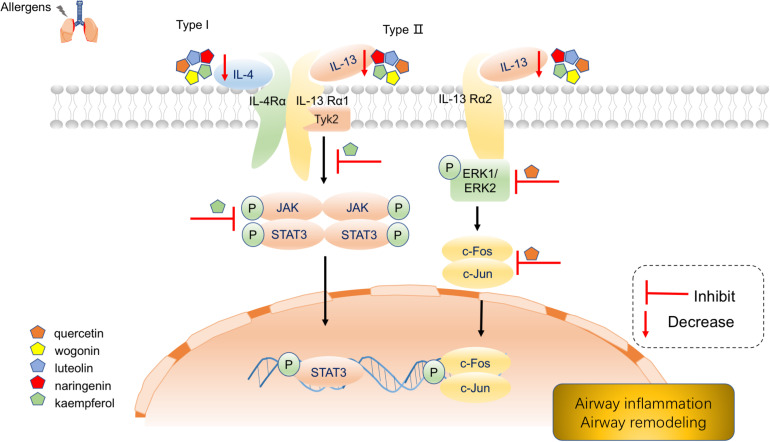
Schematic diagram depicting main components of MGMD inhibits IL-4 and IL-13 to mitigate airway allergic responses.

Our Reactome pathway enrichment results consistently argue that MGMD reduces allergic airway inflammation through the biosynthetic pathways of SPMs. Arachidonic acid metabolism, metabolism of lipids, biosynthesis of EPA-derived SPMs, biosynthesis of DHA-derived SPMs and biosynthesis of DPAn-3 SPMs were all enriched in Reactome pathway enrichment analysis. Additionally, interleukin-4 and interleukin-13 signaling, signaling by interleukins and immune system pathways were also enriched in our results. IL-4 and IL-13 are principal regulatory cytokines mainly secreted by activated Th2 cells, crucially important during the immune response in allergy and asthma (Nelms et al., 1999; Hershey, 2003). The receptors of allergic cytokines, including IL-4, IL-5, and IL-13, trigger the JAK/STAT pathway (Hsieh et al., 2011; Howell et al., 2018). Therefore, we propose that MGMD compounds targets the IL-4 and IL-13 pathway, which in turn activate transcription factors to modulate clinical symptoms of asthma (Figure 7).

According to the compound-target interaction analysis, six targets with higher degrees on the PPI network (STAT3, PTGS2, JUN, VEGFA, EGFR, and ALOX5) were identified. In our molecular docking analysis, the binding mode with the best docking score was selected to analyze the interaction between the protein receptor and its predicted active compounds. The docking scores of all compound-target pairs are lower than −5 kcal/mol, indicating that each of the five core compounds (quercetin, wogonin, luteolin, naringenin, and kaempferol) has good binding affinity to all six targets. These results suggested that the core ingredients of MGMD could relieve airway inflammation and remodeling through binding STAT3, PTGS2, JUN, VEGFA, EGFR, and ALOX5.

## Conclusion

On the ground of the above, network pharmacology strategy was presented to investigate the active compounds, potential anti-asthmatic targets and involved regulatory pathways in MGMD. Our results revealed the active ingredients and potential molecular mechanism by which MGMD treatment is effective against airway inflammation and remodeling in asthma through regulating IL-4 and IL-13 signaling and SPMs biosynthesis. These findings were partially validated previous studies. Our subsequent molecular docking showed that each bioactive compounds (quercetin, wogonin, luteolin, naringenin, and kaempferol) of MGMD has favorable binding abilities with STAT3, PTGS2, JUN, VEGFA, EGFR, and ALOX5, further arguing the potential molecular mechanism of action of MGMD in asthma. Our results will serve as a comprehensive reference for investigation of the mechanism by which MGMD intervene asthma.

## Data Availability Statement

The datasets presented in this study can be found in online repositories. The names of the repository/repositories and accession number(s) can be found in the article/Supplementary Material.

## Author Contributions

GW, BZ, and ZW: conceptualization. GW and BZ: methodology and visualization. BZ: software. GW and ZW: writing–original draft preparation. BZ, YM, YL, and XY: writing–review and editing. XY and CF: supervision and project administration. All authors have read and agreed to the published version of the manuscript.

## Conflict of Interest

The authors declare that the research was conducted in the absence of any commercial or financial relationships that could be construed as a potential conflict of interest.

## Footnotes

1 http://herb.ac.cn

2 http://www.tcmip.cn/ETCM/index.php/Home/Index/

3 http://tcmspw.com/tcmsp.php

4 http://www.swissadme.ch/

5 https://pubchem.ncbi.nlm.nih.gov/

6 http://sea.bkslab.org

7 http://ctdbase.org/

8 https://omim.org/

9 https://www.genecards.org/

10 http://string-db.org

11 http://www.cytoscape.org/

12 https://david.ncifcrf.gov/

13 https://reactome.org/

14 http://www1.rcsb.org/

## Abbreviations

AD: anti-allergic decoction
ADME: absorption distribution, metabolism and excretion
ALOX5: arachidonate 5-lipoxygenase
BP: biological process
H-C -T: herb-compound-target
DHA: docosahexaenoic acid
DL: drug-likeness
FF: Fangfeng (*Saposhnikoviae Radix*)
EPA: Eicosapentaenoic acid
GO: Gene ontology
HL: Half-life
JG: Jiegeng (*Platycodon grandiflorus*)
JUN: c-Jun NH2-terminal kinase
MGMD: Modified Guo Min decoction
MF: molecular function
OB: oral bioavailability
PPI: Protein–protein interaction
PUFA: polyunsaturated fatty acid
QH: Qianhu (*Peucedanum praeruptorum*)
SPM: Specialized pro-resolving mediator
TCM: traditional Chinese medicine
TCMSP: traditional Chinese medicine system pharmacology database
VEGFA: vascular endothelial growth factor A
WM: Wumei (*Prunus mume*)
WWZ: Wuweizi (*Schisandra chinensis*)
YCH: Yinchaihu (*Stellaria dichotoma L. var. lanceolata Bge*).
